# Changes of resistance indices after medication in benign prostatic hyperplasia: a prospective study

**DOI:** 10.1016/j.prnil.2023.02.001

**Published:** 2023-02-27

**Authors:** Dong Jin Park, Se Yun Kwon, Young Jin Seo, Hye Jin Byun, Kyung Seop Lee

**Affiliations:** a Department of Urology, Dongguk University College of Medicine, Gyeongju, Korea; b Department of Urology, Keimyung University School of Medicine, Daegu, Korea

**Keywords:** Benign prostatic hyperplasia, Resistive index, Transrectal ultrasonography, 5α-Reductase inhibitors, α-Adrenergic blockers

## Abstract

**Background:**

This study aimed to determine the relationship between resistive indices (RIs) and changes in prostate size after medical treatment in patients with benign prostatic hyperplasia (BPH).

**Methods:**

A total of 86 patients with BPH were included in the study, excluding 42 patients with a total prostate volume (TPV) of <30 cc or taking α1-adrenergic blockers and 5α-reductase inhibitors (5ARI) before study participation. Therefore, the data for 44 patients were analyzed. All patients were treated with α1-adrenergic blockers and 5ARIs. The variables examined were prostate-specific antigen, International Prostate Symptom Score, quality of life score, maximal urinary flow rate, residual urine volume, TPV, transition zone volume, and RIs of the urethral artery and left and right capsular arteries. These variables were assessed at baseline and after 3 and 6 months of treatment.

**Results:**

The mean TPV was 43.5 ± 10.9 and decreased to 35.2 ± 11.5 and 33.9 ± 9.8 after 3 and 6 months of treatment, respectively (*p* < 0.001). The mean RI of the urethral artery, right capsular artery, and left capsular artery at pretreatment did not decrease significantly. However, comparing the baseline with 3-month data, TPV at 3 months/TPV at baseline was significantly correlated with RI changes in the left capsular artery (r = 758; *P* < 0.001).

**Conclusion:**

In patients with BPH, α1-adrenergic blocker and 5ARI medications for 3 and 6 months did not result in a significant reduction in the RI of the urethral artery and both capsular arteries. Larger scale, prospective studies are needed to evaluate the relationship between TPV and RI reductions.

## 1 Introduction

Benign prostatic hyperplasia (BPH) causes moderate-to-severe lower urinary tract symptoms (LUTS) and worsens the quality of life [1, 2, 3]. Transrectal ultrasonography is commonly used in the evaluation of BPH, and the Korean clinical practice guideline also advocates its use [4]. Grayscale transrectal ultrasonography is mainly used to measure transition zone volume (TZV) and total prostatic volume (TPV), and color Doppler ultrasound is used to evaluate the structures of the prostate capsular and the urethral arteries [5, 6, 7]. The resistive index (RI), which is defined as the systolic flow velocity minus the diastolic velocity divided by the peak systolic velocity (as determined by color Doppler ultrasound), is useful for the diagnosis and follow-up of BPH [6, 7, 8, 9, 10].

Pressure flow studies have been recommended as the gold standard for diagnosing bladder outlet obstruction (BOO), but they are invasive [6]. Transition zone indices and intravesical prostatic protrusion have been proposed for the noninvasive assessment of BOO [6]. RIs are reportedly correlated with transition zone indices, maximum urinary flow rates (*Q*_max_), postvoid residual urine volumes (PVR), International Prostate Symptom Scores (IPSS), and quality of life (QoL) scores [6,9, 10, 11, 12, 13].

Studies have reported that surgical management, such as transurethral resection and transurethral vaporization of the prostate, significantly reduces RIs in patients with BPH [8,11,14,15]. α1-Adrenergic block monotherapy has been found to significantly reduce prostate RI [15]. Few studies have evaluated the changes in RIs after treatment with α1-adrenergic blocker and 5α-reductase inhibitor (5ARI) in patients with BPH patients. This study assessed the correlation between RIs and prostate volumes after combined treatment with α1-adrenergic blocker and 5ARI in patients with BPH.

## 2 Materials and Methods

This prospective study was performed with the approval of the Institutional Review Board of Dongguk University Gyeongju Hospital (IRB number: 110757-201708-HR-02-10). Informed consent was obtained from all the study participants.

**Fig. 1:**
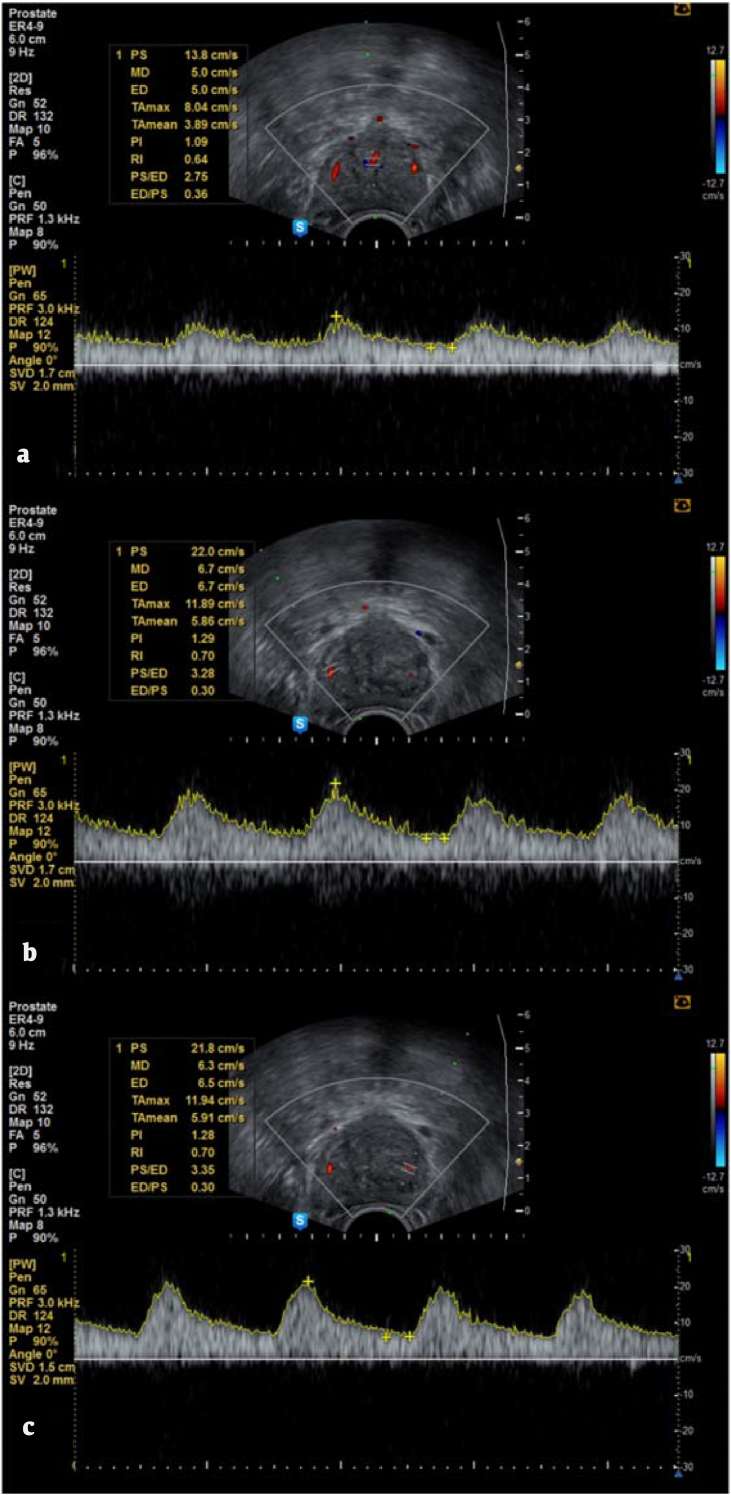
Color Doppler ultrasonogram of a patient with benign prostatic hyperplasia and resistive index measurement. (A) Urethral artery, (B) right capsular artery, and (C) left capsular artery.

Data were collected from patients diagnosed with BPH and having LUTS. A total of 86 patients were initially considered. Those with an elevated prostate-specific antigen (PSA) level (>4 ng/mL) or abnormal digital rectal examination findings underwent transrectal prostate biopsy to rule out prostate cancer. Two patients were excluded from the study for a TPV of <30 cc. The remaining 84 patients with a TPV of >30 cc were measured at baseline and compared with after 3 and 6 months of combined treatment with α1-adrenergic blocker and 5ARI. PSA levels, IPSSs, QoL scores, *Q*_max_ values, PVRs, TPVs, TZVs, and RIs of the urethral and left and right capsular arteries were also measured at baseline and compared with after 3 and 6 months of combined treatment with α1-adrenergic blocker and 5ARI. TPVs, TZVs, and RIs were measured by three urologists not otherwise involved in the study. An H60 ultrasound system (Samsung Madison, Seoul) equipped with a 6.6-MHz transrectal probe was used to measure TPVs, TZVs, and RIs (Fig. 1). Of the 84 patients, 40 who took α1-adrenergic blockers and 5ARI before the study period were excluded. Therefore, the study cohort consisted of 44 patients.

Descriptive statistics were presented as mean ± standard deviations or medians (interquartile ranges). *Q*_max_ values and PVRs at baseline and after treatments for 3 and 6 months were compared using the generalized estimating equation. Other clinical data at baseline and after treatment were compared using the generalized linear mixed model. Relationships between RI and TPV changes were analyzed using Spearman's rank correlation coefficients. All *p*-values were two sided, and *p*-values of <0.05 were considered significant. The analysis was conducted using SPSS version 26.0 (SPSS Inc., Chicago, IL).

## 3 Results

Table 1 summarizes the baseline characteristics of the 44 study participants. The mean age, body mass index, urethral artery RI, and left and right capsular artery RIs of the patients were 69.9 ± 7.5 years, 24.2 ± 2.0 kg/m^2^, 0.68 ± 0.08, 0.70 ± 0.08, and 0.68 ± 0.08, respectively. Other baseline values were as follows: the median PSA, median *Q*_max_, PVR, IPSS, QoL score, TPV, and TZV were 2.050 (1.040–3.640) ng/mL, 10.0 (6.6–13.4) mL/s, 17.0 (0.0–49.0) mL, 19.3 ± 8.1, 3.9 ± 1.0, 43.5 ± 10.9 mL, and 20.2 ± 9.3 mL, respectively. Regarding the components of α1-adrenergic blockers, tamsulosin (18, 30.9%) and alfuzosin (16, 36.4%) were the most common. For 5ARI components, dutasteride (26, 59.1%) was more common than finasteride (18, 40.9%).

**Table 1:** Baseline clinical characteristics

Variable	n = 44
Age (years)	69.9 ± 7.5
Body mass index (kg/m^2^)	24.2 ± 2.0
Hypertension, *n* (%)	21 (47.7)
Diabetes mellitus, *n* (%)	10 (22.7)
Dyslipidemia, *n* (%)	5 (11.4)
Prostate-specific antigen (ng/mL)	2.050 (1.040–3.640)
International Prostate Symptom Score	
Symptom score	19.3 ± 8.1
Quality of life score	3.9 ± 1.0
Total prostate volume (mL)	43.5 ± 10.9
Transition zone volume (mL)	20.2 ± 9.3
Maximal urinary flow rate (mL/s)	10.0 (6.6–13.4)
Postvoid residual urine volume (mL)	17.0 (0.0–49.0)
Resistive index	
Urethral artery	0.68 ± 0.08
Right capsular artery	0.68 ± 0.08
Left capsular artery	0.70 ± 0.08

Values are presented as mean ± standard deviation or median (interquartile range).

Table 2 summarizes the values of clinical variables at baseline and after 3 and 6 months of treatment. The mean IPSS at baseline and after 3 and 6 months of treatment was 19.3 ± 8.1, 12.9 ± 7.9, and 13.5 ± 8.5, respectively (*p* < 0.001 for the trend). The mean QoL scores at baseline and after 3 and 6 months of treatment were 3.9 ± 1.0, 2.6 ± 1.6, and 2.6 ± 1.5, respectively (*p* < 0.001). The mean TPVs at baseline and after 3 and 6 months of treatment were 43.5 ± 10.9, 35.2 ± 11.5, and 33.9 ± 9.8 mL, respectively (*p* < 0.001). The mean TZVs at baseline and after 3 and 6 months of treatment were 20.2 ± 9.3, 16.7 ± 8.6, 16.7 ± 8.7 mL, respectively (*p* = 0.051). The median *Q*_max_ values at baseline and after 3 and 6 months of treatment were 10.0 (6.6–13.4), 10.7 (8.5–19.5), and 12.2 (9.0–15.2) mL/s, respectively (*p* = 0.003 for the trend). The median PVRs at baseline and after 3 and 6 months of treatment were 17.0 (0.0–49.0), 12.0 (0.0–27.0), and 7.0 (0.0–35.8) mL, respectively (*p* = 0.078). The mean RIs of urethral arteries at baseline and after 3 and 6 months of treatment were 0.68 ± 0.08, 0.68 ± 0.07, and 0.67 ± 0.09, respectively (*p* = 0.873). The mean RIs of the left capsular arteries at baseline and after 3 and 6 months of treatment were 0.70 ± 0.08, 0.69 ± 0.10, and 0.67 ± 0.08, respectively (*p* = 0.212) and those of the right capsular arteries were 0.68 ± 0.08, 0.66 ± 0.09, and 0.69 ± 0.08, respectively (*p* = 0.328).

**Table 2:** Changes in clinical variables in baseline and after 3 and 6 months’ treatment using α1-adrenergic blockers and 5α reductase inhibitors

Variable	Baseline	3 months	6 months	*P*
IPSS				
Symptoms score	19.3 ± 8.1^a^	12.9 ± 7.9^b^	13.5 ± 8.5^a,b^	<0.001^d^
QoL score	3.9 ± 1.0^a^	2.6 ± 1.6^b^	2.6 ± 1.5^a,b^	<0.001^d^
TPV (mL)	43.5 ± 10.9^a^	35.2 ± 11.5^b^	33.9 ± 9.8^a,b^	<0.001^d^
TZV (mL)	20.2 ± 9.3	16.7 ± 8.6	16.7 ± 8.7	0.051^d^
*Q*_max_ (mL/s)	10.0 (6.6–13.4)^a^	10.7 (8.5–19.5)^a^	12.2 (9.0–15.2)	0.003^e^
PVR (mL)	17.0 (0.0–49.0)	12.0 (0.0–27.0)	7.0 (0.0–35.8)	0.078^e^
RI				
Urethral artery	0.68 ± 0.08	0.68 ± 0.07	0.67 ± 0.09	0.873^d^
Right capsular artery	0.68 ± 0.08	0.66 ± 0.09	0.69 ± 0.08	0.328^d^
Left capsular artery	0.70 ± 0.08	0.69 ± 0.10	0.67 ± 0.08	0.212^d^

Values are presented as mean ± standard deviation or median (interquartile range).

IPSS, International Prostate Symptoms Score; PVR, postvoid residual urine volume; *Q*_max_, maximal urinary flow rate; QoL, quality of life; RI, resistive index; TPV, total prostate volume; TZV, transition zone volume.

^a, b, c^Means or medians in columns with different superscript indicate significant different (*p* < 0.05).

d These *p-*values were calculated by generalized linear mixed model.

e These *p*-values were calculated by generalized estimating equation.

**Fig. 2:**
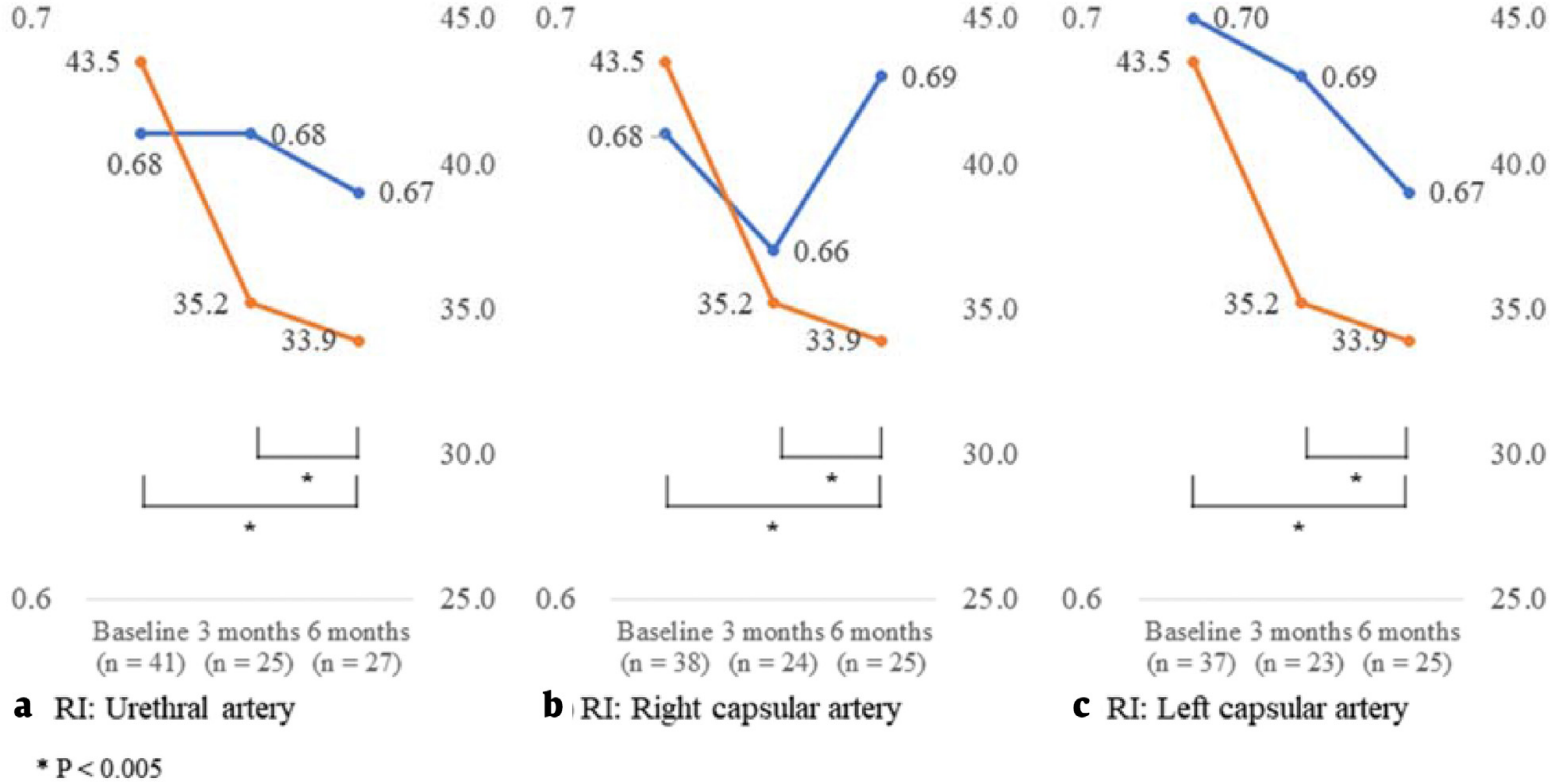
RI and total prostate volume changes at baseline and after 3 and 6 months of treatment using α1-adrenergic blockers and 5α-reductase inhibitors. RI, resistive index.

Fig. 2 demonstrates the RI changes and prostate size over time as a graph. IPSS symptom scores, QoL scores, and TPV exhibited significant changes after 6 months of treatment and between 3 and 6 months. *Q*_max_ values were changed significantly after 3 months of treatment. Other clinical variables did not exhibit significant changes posttreatment.

Table 3 summarizes the correlation coefficients between the RI and prostate volume–related variables. The baseline and 3-month values were compared and showed TPV at 3 months/TPV at baseline was significantly correlated with RI changes of the left capsular artery (r = 758; *P* < 0.001). However, no correlation was observed between the RI of the urethral and right capsular arteries and TPV at 3 months/TPV at baseline.

**Table 3:** Correlation coefficients between the RIs and prostate volume–related variables

(a) Difference from baseline to 3 months						
Variable	RI of urethral artery	*P*	RI of right capsular artery	*P*	RI of left capsular artery	*P*
TPV (mL)	−0.084	0.710	−0.088	0.711	−0.702	0.001
TZV (mL)	−0.207	0.355	−0.184	0.436	−0.378	0.122
TPV at 3 months/TPV at baseline	0.117	0.604	0.038	0.875	0.758	<0.001

(b) Difference from baseline to 6 months						

Variable	RI of urethral artery	*P*	RI of right capsular artery	*P*	RI of left capsular artery	*P*

TPV (mL)	−0.059	0.783	0.222	0.334	−0.163	0.492
TZV (mL)	−0.173	0.420	0.232	0.313	0.054	0.820
TPV at 6 months/TPV at baseline	0.045	0.834	−0.126	0.586	0.226	0.338

(c) Difference from 3 months to 6 months						

Variable	RI of urethral artery	*P*	RI of right capsular artery	*P*	RI of left capsular artery	*P*

TPV (mL)	−0.017	0.948	−0.079	0.779	0.104	0.713
TZV (mL)	0.001	0.996	−0.495	0.060	−0.094	0.740
TPV at 6 months/TPV at 3 months	−0.206	0.428	−0.002	0.993	−0.136	0.629

RI, resistive index; TPV, total prostate volume; TZV, transition zone volume.

## 4 Discussion

The present study revealed that combined treatment with α1-adrenergic blocker and 5ARI did not reduce RIs in patients with BPH. However, TPV at 3 months/TPV at baseline was significantly correlated with RI changes of the left capsular artery. Testosterone and dihydrotestosterone promote growth in the stromal and epithelial cells of the prostate gland in BPH [16]. This inner growth and the outer prostate capsule surround the prostate gland, increase intraprostatic pressure, and provoke periurethral compression [11,16]. A decrease in elasticity and the amount of collagen in the prostatic urethra reduce prostatic urethra compliance and increase resistance to flow [16,17]. These two mechanisms exacerbate BOO [16,17]. Urethral arteries, which originate from the inferior vesical arterial system, form a right angle and surround the prostate gland through the bladder neck. The capsular arteries originate from the prostatic arteries as they pass along the anterolateral surface of the prostate [7,12]. Elevated intraprostatic pressure increases prostate vascular resistance by compressing the blood vessels in the prostate, and RIs provide a measure of this increase in vascular resistance [9,11,13]. RIs may be considered as noninvasive measures of BOO severity for this reason [6,9,10,12].

α1-Adrenergic blockers loosen smooth muscle tone in the urethra, bladder neck, and prostate gland by inhibiting α1-adrenergic receptors, which predominate in these areas [18]. Bulut et al. [15] reported that alfuzosin 10 mg once daily for 3 months decreased the RIs of the prostate and capsular arteries from 0.73 ± 0.1 to 0.70 ± 0.1 (*p* = 0.0001) in patients with mild-to-moderate LUTS and *Q*_max_ values of <15 mL/s. Significant RI reductions of prostate capsular arteries after treatment with α1-adrenergic blocker imply that prostate muscle tone may be associated with intraprostatic pressure [15]. However, no significant difference was observed between RIs at baseline and after 3 months of treatment in patients with TPVs of ≥30 cc who had received a combined treatment of α1-adrenergic blocker and 5ARI. Previous reports have shown that α1-adrenergic blocker monotherapy is most effective at 3 months in BPH with TPVs ≥30 cc, as determined using IPSSs [19], and 5ARI monotherapy improves symptoms after more than 4–6 months of treatment and reduces TPVs and TZVs continuously until 24 months [20,21]. α1-Adrenergic blocker monotherapy exhibited clinical progression in patients as TZVs increased over time [19,20]. These findings show that decreases in intraprostatic pressures induced by α1-adrenergic blocker monotherapy were insufficient in these patients. However, doses and types of α1-adrenergic blockers were not unified in these studies, and these two factors may have contributed to this result.

The baseline RIs of the prostate capsular arteries of this study were lower than those previously reported (0.68–0.70 vs. 0.75–0.79) [8,14,15]. However, these studies included patients who underwent surgical treatment and had larger TPVs (43.5 vs. 62.5–65.7 mL), PVRs (17.0 vs. 77.4–139.9 mL), higher IPSS scores (19.3 vs. 18–25.3), and lower *Q*_max_ values (10.0 vs. 6.9–8) [8,14,15]. If we consider that a correlation exists between RIs and the severity of BOO, the difference between BOO severity in patients who underwent medical or surgical treatment might explain the observed baseline differences between the RIs of prostate capsular arteries [6].

The longitudinal changes in prostate capsular RIs observed in this study were smaller than those reported by other studies that included patients who underwent surgical treatment. Although intraprostatic pressure starts decreasing from 1 month after surgical treatment, the effect of 5ARI treatment on TPV continues for 24 months [8,20,21]. Patients were followed after treatment only for 6 months, which was insufficient to observe the effect of 5ARI. Bulut et al. [15], in a comparative study of medical and surgical treatment groups at 3 months, reported that the surgical treatment group showed greater reductions in the RIs of prostate capsular arteries, which suggests α1-adrenergic blocker monotherapy–induced reductions in RIs of prostate capsular arteries are less than those achieved by surgical treatment [15]. A shorter follow-up period and different treatment modalities may explain the observed differences between the RIs of the prostate capsular arteries.

The RIs of urethral arteries did not decrease significantly after the combined treatment with α1-adrenergic blocker and 5ARI in the present study. Tsuru et al. [22] reported that the RIs of urethral arteries were not correlated with TPV, TZV, IPSS, and *Q*_max_. RIs of prostate capsular arteries were taken as assessment tools in the majority of studies that have evaluated changes in RIs in BPH after treatment [8,14,15]. 5ARI reduces TZVs and TPVs [20,21], and the associated gradual decrease in intraprostatic pressure may be associated with decompression of urethral and prostate capsular arteries.

Although RIs of the urethral and right capsular arteries were not significantly correlated, the only significant finding was that TPV at 3 months/TPV at baseline was significantly correlated with RI changes of the left capsular artery. Previous studies demonstrated that TPV was correlated with RI in BPH [8,12]. To the best of our knowledge, only a few studies reported the correlation between TPV and RI changes. Considering that the greater the rate of decrease in TPV, the greater the decrease in prostatic pressure, these results may have some significance [9,11,13]. Because RI on the opposite side was not correlated with TPV at 3 months/TPV at baseline, further studies are needed to confirm this clinical significance.

This was believed to be the first prospective study to compare the RIs of urethral arteries and left and right capsular arteries at baseline and after 3 and 6 months of combined treatment with α1-adrenergic blocker and 5ARI in patients with BPH. The study results demonstrated nonsignificant treatment-induced RI changes in the urethral and both capsular arteries. Approximately 50% of patients had missing values during follow-up periods, which would be one of the reasons that this study demonstrated nonsignificant results.

Also, this study has several limitations. First, the sample size was small. Second, lifestyle factors (e.g., alcohol consumption and cigarette smoking) were not considered, which affect LUTS and vascular diseases [15,23, 24, 25]. Third, as mentioned earlier, the study period was not long enough to access the effect of 5ARI. Fifth, the study did not contain a control group (e.g., α1-adrenergic blocker alone, 5ARI alone, or a placebo group).

## 5 Conclusions

It was shown that the combined treatment with α1-adrenergic blocker and 5ARI for 3 and 6 months did not significantly reduce the RIs of urethral and both left and right capsular arteries in BPH patients with a TPV of ≥30 cc. Larger scale, prospective studies, including the control group, are required to evaluate the nature of the relationship between TPV reduction and RIs after the combined treatment.

## Conflict of interest

The authors have no conflict of interest to declare.
